# Remotely detuned receiver coil for high-resolution interventional cardiac magnetic resonance imaging

**DOI:** 10.3389/fcvm.2023.1249572

**Published:** 2023-10-30

**Authors:** Sina Marhabaie, Marylène Delcey, Dounia El Hamrani, Fanny Vaillant, Jean-Christophe Ginefri, Valéry Ozenne, Emma Abell, Marie Poirier-Quinot, Bruno Quesson

**Affiliations:** ^1^ Laboratoire D'Imagerie Biomédicale Multimodale Paris Saclay, Université Paris-Saclay, CNRS, Inserm, Orsay, France; ^2^ Univ. Bordeaux, INSERM, CRCTB, U 1045, IHU Liryc, Bordeaux, France; ^3^ Siemens Healthineers, Saint-Denis, France; ^4^ Univ. Bordeaux, CNRS, CRMSB, UMR 5536, IHU Liryc, Bordeaux, France

**Keywords:** MRI, intervention, coil design, MR-thermometry, heart, detuning, catheter

## Abstract

**Introduction:**

Interventional cardiac MRI in the context of the treatment of cardiac arrhythmia requires submillimeter image resolution to precisely characterize the cardiac substrate and guide the catheter-based ablation procedure in real-time. Conventional MRI receiver coils positioned on the thorax provide insufficient signal-to-noise ratio (SNR) and spatial selectivity to satisfy these constraints.

**Methods:**

A small circular MRI receiver coil was developed and evaluated under different experimental conditions, including high-resolution MRI anatomical and thermometric imaging at 1.5 T. From the perspective of developing a therapeutic MR-compatible catheter equipped with a receiver coil, we also propose alternative remote active detuning techniques of the receiver coil using one or two cables. Theoretical details are presented, as well as simulations and experimental validation.

**Results:**

Anatomical images of the left ventricle at 170 µm in-plane resolution are provided on *ex vivo* beating heart from swine using a 2 cm circular receiver coil. Taking advantage of the increase of SNR at its vicinity (up to 35 fold compared to conventional receiver coils), real-time MR-temperature imaging can reach an uncertainty below 0.1°C at the submillimetric spatial resolution. Remote active detuning using two cables has similar decoupling efficiency to conventional on-site decoupling, at the cost of an acceptable decrease in the resulting SNR.

**Discussion:**

This study shows the potential of small dimension surface coils for minimally invasive therapy of cardiac arrhythmia intraoperatively guided by MRI. The proposed remote decoupling approaches may simplify the construction process and reduce the cost of such single-use devices.

## 1. Introduction

In common clinical situations, the receiver coils of an MRI are positioned around the patient near the area of interest to be imaged (1). In addition to diagnosis, MRI-guided interventions are increasingly being used since real-time imaging can be exploited to interactively position a therapeutic device near the region to treat and then monitor the therapy (2). Illustrative examples have been reported for various therapeutic devices including high-intensity focused ultrasound (3, 4), radiofrequency (5), laser (6), and microwave (7) ablations in various organs (e.g., brain, uterine fibroid, prostate, liver, pancreas, etc.). Most therapeutic devices are positioned percutaneously or through vascular access (e.g., in the heart) to deliver therapeutic energy a few millimeters/centimeters around their active tip. For such applications, a high signal-to-noise ratio (SNR) is desirable in this area to precisely define the targeted pathologic tissue and to spatially and temporally control energy deposition to selectively alter pathologic tissues and preserve surrounding healthy tissues. Thus, a small-dimension MRI receiver coil may provide a significant gain in SNR compared to conventional external coils of large dimensions and allow a reduction of the field of view of the images (8), taking advantage of its intrinsic spatial selectivity. Gain in SNR can thus be exploited to improve the spatial resolution of the images and/or to reduce acquisition time.

However, receiver coils must be detuned (or decoupled; in this concept, these two words have been used interchangeably in the literature) during the emission of the *B*_1_ field by the transmitting coil. Otherwise, a current is induced in the receiver coil and creates an additional local *B*_1_ field around the receiver coil that will locally affect the contrast on the images. In addition, this induced current can heat and eventually burn both the surrounding tissues and the coil, creating a hazardous situation for the patient (9). These problems can be overcome by resorting to a resonant blocking circuit that effectively impedes current circulation during *B*_1_ transmission. Such blocking circuits are usually LC resonant circuits with the same resonance frequency as the receiver coil. They are activated (during *B*_1_ radiofrequency pulses) and deactivated (otherwise) synchronously with the MRI acquisition sequence using diodes (typically PIN diodes), and are usually labeled as “active detuning”. The aforementioned classic method works fine in ordinary situations and results in images with no (or imperceptible) *B*_1_ artifacts if the detuning efficiency is sufficient.

Active detuning of receiver coils inserted percutaneously becomes challenging and creates concerns about patient safety. For instance, in intravascular coils used in intra-cardiac MRI, the DC applied for activating the PIN diode for detuning the intracavitary coil may induce a cardiac arrest in case of an electrical leakage above 50 mA (e.g., a contact between the diode and the myocardium). Furthermore, although miniaturization of the components could be a potential solution, assembling the required electronic circuits in the limited space of an intravascular catheter requires sophisticated technologies. Such an approach has been proposed in the context of catheter-based radiofrequency ablation of atrial fibrillation, where a sheath embedding the ablation catheter was equipped with a deployable MR-receiver coil (10). Other methods have been proposed for imaging vessel walls using intravascular coils (11), but sometimes with a limited technical description of detuning techniques (12), compromising the clinical applicability of such devices for interventional cardiac MRI. Altogether, this increases the complexity and construction costs that must be kept at a reasonable level for single-use disposable devices. In such cases, a practical strategy is to position the electrical components at a distance from the tip of the device. This approach has already been performed to adjust the frequency (tune) and the impedance (match) of the MR coils (13, 14). Despite all its potential applications (ensuring high-quality images and patient safety), remote detuning of receiver coils has not been duly investigated in the literature. Note that, positioning the detuning circuit outside the patient's body by simply connecting its components via a piece of cable (or other conductors) is ineffective because this will degrade the radiofrequency (RF) properties of the coil (notably its *Q* factor) and hence its sensitivity, reducing the resulting SNR of the images.

The present work has been carried out as the first step toward the realization of a deployable MRI receiver coil embedded in an intracardiac catheter to obtain high-resolution cardiac MR images (10, 13). Some potential applications could be better diagnosis and treatment monitoring in patients suffering from atrial or ventricular arrhythmia, exploiting high-resolution MRI (10) of the cardiac substrate to better characterize the tissue to ablate, real-time monitoring of the therapy (15, 16), and immediate post-ablation 3D imaging (16, 17) to assess the therapeutic success of the procedure.

The study has a two-fold objective:

- First, we illustrate the potential of such a local receiver coil in the context of interventional cardiac MRI for the treatment of cardiac arrhythmia. We show the gain in spatial resolution provided by a 2 cm diameter circular coil for high-resolution cardiac imaging of ex vivo beating swine heart and for improving precision and spatial resolution of real-time MR-thermometry. Such a coil dimension is well fitted to the internal dimensions of the cardiac chambers. As an example, clinical therapeutic devices such as cryo-ablation balloons (Artic Front, Medtronic) designed for the treatment of atrial fibrillation range similar size (25 mm in diameter).
- Second, we present innovative remote detuning technologies to provide a safe, efficient, and cost-effective solution for receiver coil detuning. Our theoretical work is supported by circuit simulations and experimental validation.

## 2. Materials and methods

**Figure 1:**
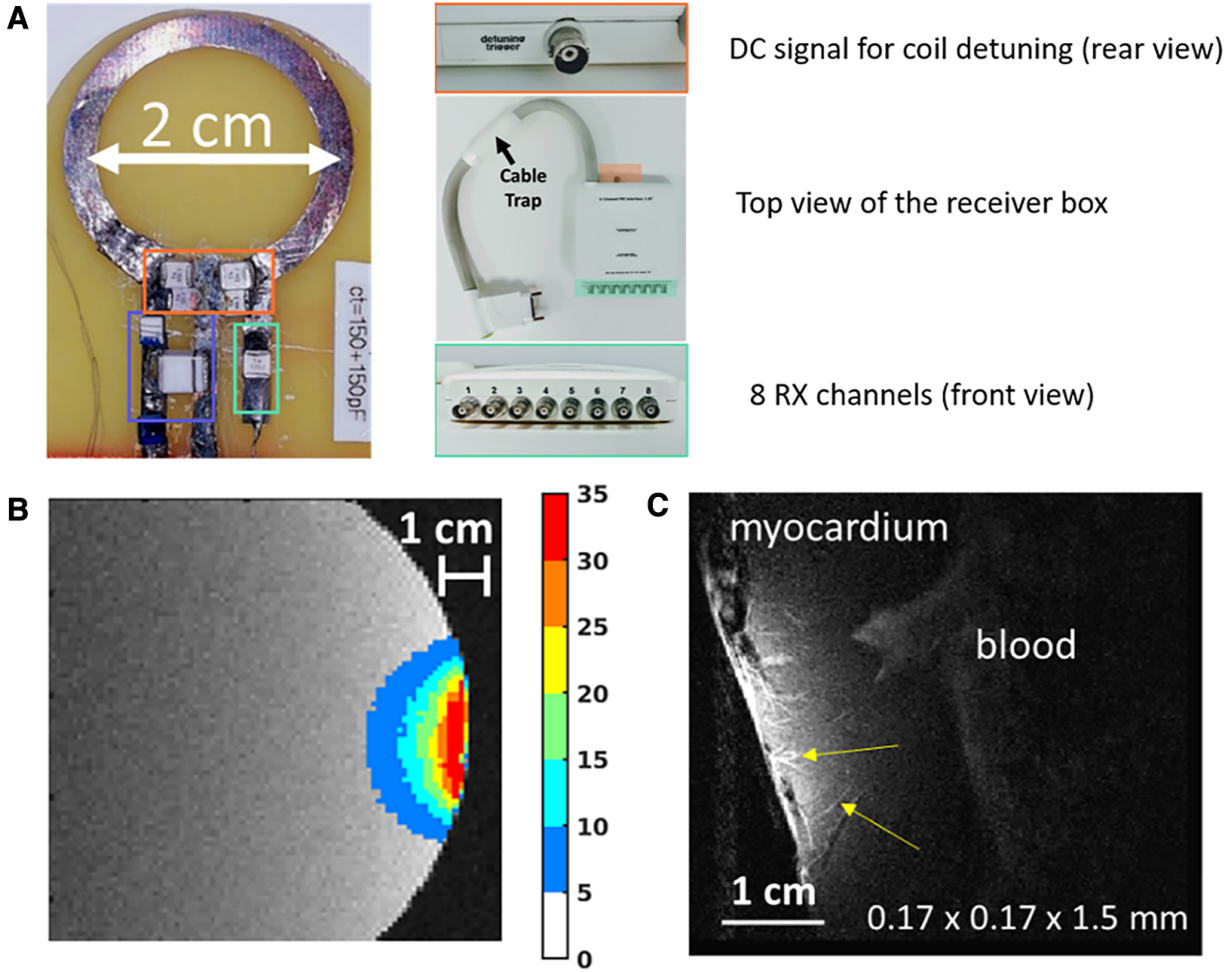
Illustrations of the expected MR-image improvement using a small diameter circular surface coil for high-resolution cardiac MRI. (**A**) Photograph of the implemented coil of 2 cm diameter and interface box for connecting to the MRI scanner. Orange, green, and blue rectangles indicate tuning capacitors, matching capacitors, and active decoupling circuits, respectively. (**B**) Measurement of the gain in signal-to-noise ratio provided by the developed coil relative to the manufacturer's coil, measured on a phantom. (**C**) Gradient-echo image acquired at 170 µm in-plane resolution and 1.5 mm slice thickness on an ex vivo beating heart from a pig. Microvasculature within the myocardium can be observed in hyper signals (yellow arrows).

To evaluate the potential gain of a local loop coil for interventional cardiac MRI, we implemented a circular loop of 2 cm diameter with conventional on-site active detuning using a PIN diode (see Figure 1A). A custom-built 8-channel receiver box (STARK contrast MRI Coil Research, Germany) interconnected the receiver coil with the scanner (see Figure 1A, right). This box was used to receive the MR signal of our homemade coils while providing a DC signal synchronized with the pulses of any MRI sequence to detune the receiver coil during RF emission by the body coil. Two 50 *Ω* coaxial cables were used for both signal reception and routing the DC signal to the PIN diode. The cables were lying on the table and were roughly aligned with B_0_. In order to test the effectiveness of coil detuning, we connected the coil to the interface box (receiving channel and DC connector) and turned it off in the IRM interface (detuned coil), while enabling the manufacturer's coils for MRI signal detection.

### 2.1. Quantification of the gain in SNR

The gain in SNR provided by the loop coil was measured on a phantom (3.75 g NiSO_4 _× 6H_2_O + 5 g NaCl per liter in demineralized water, provided by the MRI manufacturer) by computing the SNR ratio between two images acquired with identical parameters (multislice 2D gradient echo images: FOV = 200 × 200 mm; Matrix size = 192 × 192 pixels; TR/TE = 1,784/5 ms; FA = 15°; bandwidth = 300 Hz/pixels; 1 × 1 × 2.5 mm^3^ voxel size), the first one being acquired with the Loop coil and the second one with the conventional clinical 18 elements chest coil plus the 4 elements spine coil. The manufacturer's array coil was positioned 4 cm from the phantom to replicate the typical spatial layout of a clinical cardiac MRI with the array surrounding the patient's thorax. The 2 cm surface coil was in contact with the phantom to approximate the final configuration expected of an intracavitary coil.

### 2.2. Experiments on ex vivo beating swine hearts

To estimate the achievable spatial resolution of cardiac MRI using the loop coil, we performed experiments on ex vivo beating hearts from swine (*N* = 3). The protocol was approved by the Animal Research Ethics Committee of Bordeaux, France (CEEA50). Each animal (Large White × Landrace, ∼40 kg) was premedicated with ketamine (20 mg.kg^−1^) and acepromazine (1 mg.kg^−1^) injected IM. Induction of anesthesia was realized with intravenous bolus of ketamine (15 mg.kg^−1^) and midazolam (1.5 mg.kg^−1^). After induction of anesthesia, the animal was intubated and ventilated and received an injection of heparin (2.5 mg.kg^−1^). Anesthesia was maintained with ketamine and midazolam (40 mg.kg^−1^.h^−1^ and 2 mg.kg^−1^.h^−1^ respectively). The thorax was surgically opened, and blood from the animal was collected (∼3 L). Cardiac arrest was realized by cross-clamping of the ascending aorta and direct injection in the aortic root of 1 L of cold (4°C) cardioplegic custodiol solution, before a rapid excision and immersion in a cold 0.9% saline solution (18, 19). The aorta, and pulmonary artery were then cannulated. After the heart was prepared, it was reperfused in the Langendorff mode for 15–20 min to wash out the cardioplegic solution, gradually rewarm the heart, and recover a stable ex vivo cardiac function. Perfusion of the heart was ensured by the autologous blood collected, diluted with a Tyrode buffer (vol/vol: 1/3, 38°C) (20). Using a small homemade intraventricular catheter that was inserted in the left ventricle via the apex and connected to a fluid-filled piezoelectric pressure transducer, located outside the Faraday cage, for continuous monitoring of left ventricular pressure (LabChart 8.0 software, ADInstrument, Oxford, United Kingdom) and to generate a TTL trigger used to synchronize the MR acquisition. Dedicated homemade harm maintained the coil in contact with the left ventricle. Description of the experimental setup is given in Supplementary Material Figure S2).

### 2.3. MR-thermometry experiments in a gel phantom

Using the loop coil, we also investigated the precision and improvement of spatial resolution of MR-temperature imaging on gel under well-controlled heating conditions. MR-thermometry was performed using the proton resonance frequency shift method on a gel. The acquisition sequence was a single-shot gradient-echo echo planar imaging (EPI) sequence, with the following parameters: FOV = 123 × 123 mm^2^, TR/TE = 1,000/23 ms, matrix size = 78 × 78 (zero filled to 156 × 156) with slice thickness = 3 mm for a spatial resolution of 1.6 × 1.6 × 3 mm^3^, bandwidth = 1,455 Hz/pixel, FA = 53°, GRAPPA acceleration factor = 2, 7/8 Partial Fourier. The acquisition was first performed with a flexible array composed of 4 elements (FL, provided by the manufacturer) and the Spine (SP, 2 coils with 4 elements each) coils. In a second experiment, these coils and the loop coil taped on the side of the gel were used for image reconstruction (13 receiver elements in total). For each experiment, 120 dynamics were acquired. For both configurations, phase images were processed to compute temperature maps (using a homemade Matlab code) during radiofrequency energy deposition (15 W power emitted during 40 s) using an MR-compatible catheter (Imricor Medical Systems, Burnsville, Minnesota, USA) inserted vertically into the gel. Raw and low-pass filtered (first order Butterworth with 0.04 Hz cutoff frequency) temperature curves at the hottest pixel were plotted. Temporal standard deviation (*σ*T) of temperature before heating, together with maximal ± *σ*T at the end of energy deposition were computed to compare the precision of thermometry for each experimental condition.

In a separate batch of experiments, a laser optic fiber (975 nm, LuOcean Mini 4, Lumics, Berlin, Germany) was inserted in the gel to create a small hot spot (0.4 W power emitted during 2 min 24 s) under MR-thermometry using only the Loop coil taped on the side of the gel. This heating configuration was chosen to illustrate the effects of partial volume on temperature measurement when a coarse spatial resolution is used relative to hotspot dimensions. We acquired four sets of MR-temperature images (single shot EPI, TR/TE = 340/38 ms, bandwidth = 1,455 Hz/pixel, FA = 53°, and 7/8 Partial Fourier, no grappa acceleration, 500 dynamic acquisitions) at different spatial resolutions by adjusting the FOV (100 × 100, 61 × 61, 61 × 61, and 53 × 53 mm), the matrix size (64 × 64, 64 × 64, 64 × 64, and 76 × 76), and the slice thickness (3, 3, 2 and 2 mm). Identical image processing was applied to images, including temporal low-pass filtering on a pixel-by-pixel basis, as described above. The temporal standard deviation (*σ*T) of temperature was computed for each pixel in a region of interest of ∼12 × 12 mm^2^ around the laser tip position over the 20 first acquisitions preceding heating. The maximal temperature increase ± *σ*T was also computed.

### 2.4. Remote detuning implementations

**Figure 2:**
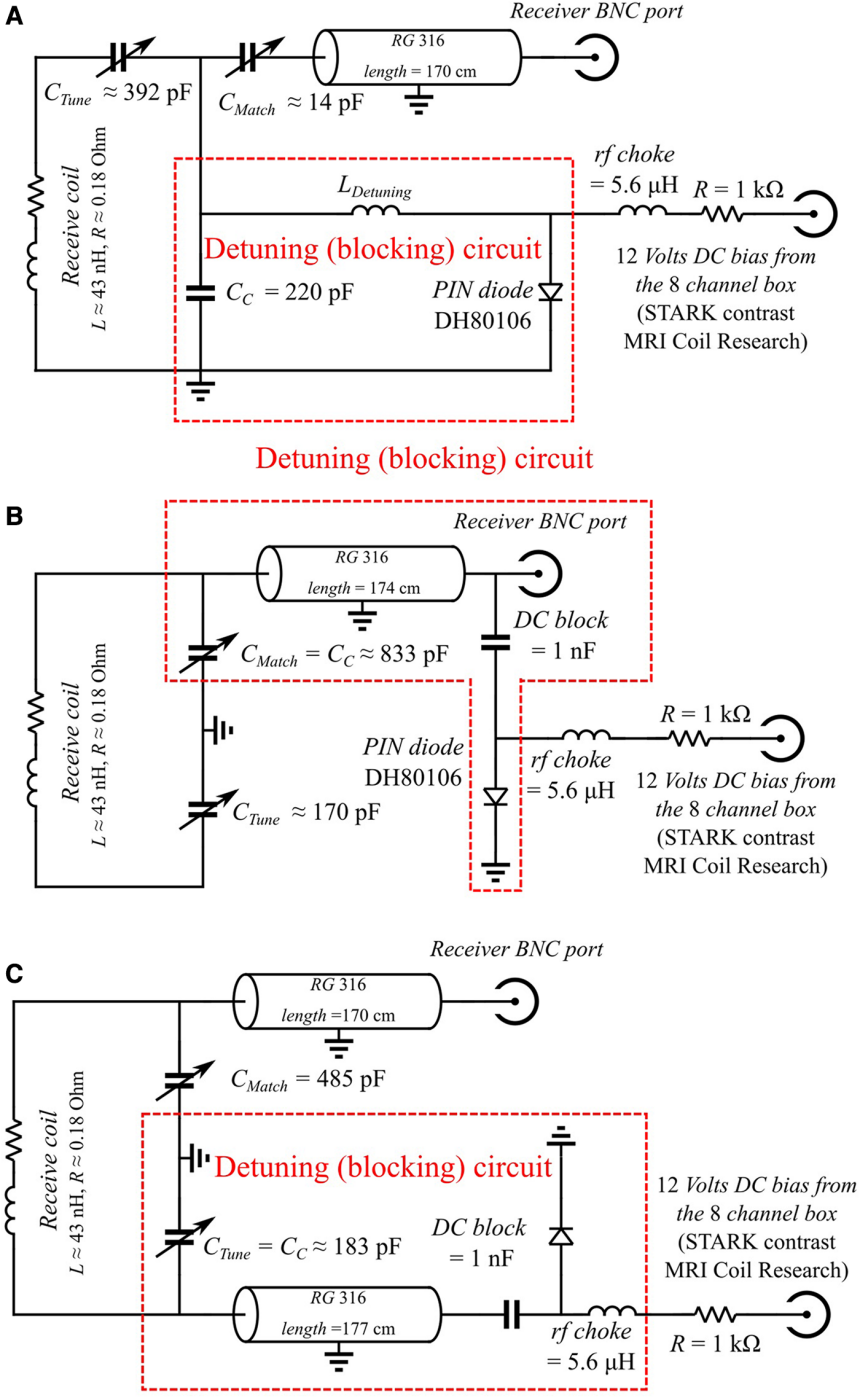
Circuit schematic diagrams for active detuning of the receiver coils. Values of the components are indicated. In all cases, the receive coil was a circular loop of diameter 2.0 cm etched on a standard printed circuit board. (**A**) Schematic of conventional on-site detuning using a PIN diode near the receiver coil. (**B**) Schematic of remote detuning using one cable. The coaxial cable shorted at the end, serves as a short-end stub. The length of the coaxial cable is chosen such that its equivalent inductance L_Stub_ forms a resonant circuit with C_Match_ at the Larmor frequency *f*_0_. In this circuit, C_Match_ matches the receive coil to the line's impedance (usually 50 Ohms) and couples the receive coil to the blocking circuit. (**C**) Schematic of remote active decoupling using two cables. When the diode is not powered (receive phase, blocking circuit is open), receive coil resonates at its original frequency *f_0_*. When the diode is powered (transmit phase, blocking circuit is closed), the coaxial cable in the bottom is shorted at the end and serves as a short-end stub. The bottom coaxial cable length is chosen carefully to form a resonant circuit with C_Tune_. In this circuit, C_Tune_ is used to both resonate the receive coil at the Larmor frequency and couple the receive coil to the blocking circuit.

In this part, we compare three methods for active detuning, using conventional on-site detuning using a PIN diode positioned near the receiver coil (Figure 2A), and two remote active detuning methods using either one cable (Figure 2B) or two cables (Figure 2C) to create a blocking circuit during RF excitation. The principle of the detuning in all these three methods is to couple the RF coil to a second resonant circuit (the blocking circuit) also tuned at the Larmor frequency *f_0_* during the transmission. Considering the general properties of two coupled oscillators (21), once the two circuits are coupled together, they behave as a new system with two distinct resonance frequencies, *f_+,_* and *f_−_*. If these two frequencies are far enough from the original *f_0_*, the coil will show a large impedance at *f_0_*, and therefore, current circulation will be effectively prohibited during transmission. The quality of the detuning circuit is evaluated through two parameters: the Detuning Efficiency (*DE*) and the Detuning Offset (*DO*). The detuning efficiency (measured in dB units) is defined as the difference between the *S*_12_ values of the non-detuned and the detuned coils at the resonance frequency *f_0_* (described in the annex in the Supplementary Material) and is measured using a standard double-loop probe *S*_12_ measurement (22, 23). The detuning offset is defined as *DO* = min (|*f_+/−_*–*f_0_*|). The more the frequency offset (concerning the resonance frequency), the larger the impedance at *f_0_*, and the more efficient the detuning (larger *DE*).

#### 2.4.1. Principles of remote detuning using one coaxial cable

In their article, Edelstein et al. (24) proposed to use a piece of coaxial cable as the inductor of the blocking circuit (Figure 2B). During the receive phase, the termination of the coaxial cable is open and serves as a signal carrier whereas during the transmit phase the coaxial cable is shorted and serves as a short-end stub. Note that there is only one cable for signal carrying and active detuning, so the cable must be connected to the matching capacitor C_Match_ to couple the receive coil to the blocking circuit. However, one cannot freely choose C_Match_ such that the detuning offset is large enough (see annex in the Supplementary Material) to obtain an efficient detuning since its value is imposed by the impedance of the signal-carrying cable (usually 50 Ohms) and its length. Moreover, the detuning efficiency depends also on the energy losses in the blocking circuit, which depends on the thickness of the cable conductor, the nature and dielectric constant of the insulating material, and the length of the cable.

#### 2.4.2. Remote detuning using two coaxial cables

Another possible implementation of remote detuning is depicted in Figure 2C, where one cable is dedicated to carrying the MRI signal, and another cable is used for active detuning. C_C_ is the coupling capacitance that allows coupling the RF coil with the decoupling circuit, C_Match_ the matching capacitance to ensure present a 50 Ohms equivalent impedance to the signal-carrying cable. Such an approach provides more flexibility to choose a sufficiently small value for C_C_ such that the detuning offset is large enough to reach a satisfactory detuning efficiency (see annex in the Supplementary Material).

#### 2.4.3. Simulations of the different active detuning circuits

The three detuning configurations in Figure 2 were simulated using the QucsStudio (25) (version 3.3.2). Frequency responses were obtained by exciting the circuit with an alternating voltage source and simulating the current passing through the voltage source. The coil inductance was calculated using the “Component Designer” module of QucsStudio. All types of energy loss in the receiver coil were simulated by a resistance *R* in series with the coil. The *Q* of the coil was measured on the test bench to compute this resistance (R = L*ω*/*Q*_unloaded_). Since the same inductor was used for all three coils, the resistance and inductance values were assumed to be identical whatever the detuning strategy.

#### 2.4.4. Implementation and characterization of actively detuned receiver coils

Three circular receiver coils of 2 cm in diameter were implemented and corresponded to each circuit shown in Figure 2, etched on a standard printed circuit board (PCB FR4, copper thickness = 35 µm, board thickness = 1 mm). Appropriate ceramic capacitors (Exxelia, non-magnetic) were systematically used to resonate the receiver coil at the Larmor frequency and to match the circuit to 50 Ohms.

To determine the resonance frequency and the *DE* value, standard double-loop probe measurements (22, 23) were carried out by using a Rohde & Schwarz vector network analyzer (VNA, model ZNLE3) and a homemade double-loop probe. To include the loading effects, measurements were performed in the presence of a cylindrical plastic bottle of diameter ca 95 mm, filled with a solution of 0.770 g CuSO_4_.5H_2_O + 2.0 g NaCl + 1 ml arquad (1% solution) + 0.05 ml H_2_SO_4_, (0.05 M solution) in 1,000 ml demineralized water.

The sensitivity of each receiver coil and efficiency of detuning was also investigated, performing MRI experiments on a 1.5 T AERA scanner (Siemens Healthineers, Germany). The body coil was systematically used for RF excitation. Each coil was taped on a phantom and positioned inside a volume head coil from the manufacturer (Siemens Tim Coil, 20-element array, Head/Neck20). Multislice 2D gradient-echo images were obtained using the following parameters: field of view = 200 mm × 200 mm, matrix size = 192 (read) × 192 (phase), echo time = 5.84 ms, repetition time = 11.23 ms, flip angle = 7 degrees, number of repetitions = 1, and slice thickness = 4.5 mm. To compare the images of different scans, the images were converted to SNR maps by dividing the signal of each pixel by the standard deviation of the noise in each image using the software ImageJ (26).

## 3. Results

Figure 1 illustrates the potential of using a 2 cm diameter circular coil (using conventional on-site decoupling, Figure 1A) in the context of high-resolution cardiac MRI. Figure 1B illustrates the gain in SNR compared to conventional coils, with a maximal gain of around 35 (measured at the very close proximity of the coil over a region of 2 × 1.5 cm^2^), and an improvement in SNR higher than 20 up to 1 cm distance from the coil. Figure 1C displays an example of a 2D slice acquired on the left ventricle of a beating swine heart (96 ± 3 bpm) using a gradient echo sequence triggered in the diastolic phase (FOV = 50 × 50 mm^2^; Matrix = 290 × 290 pixels; TR/TE = 593/25 ms; FA = 60°; bandwidth = 130 Hz/pixels, 1 k-space segment acquired per cardiac contraction, 2 min 30 s acquisition time) with an in-plane spatial resolution of 170 µm and a slice thickness of 1.5 mm. Submillimeter vessels can be visualized within the myocardium (Other representative examples of high-resolution cardiac imaging are provided in the Supplementary Materials).

**Figure 3:**
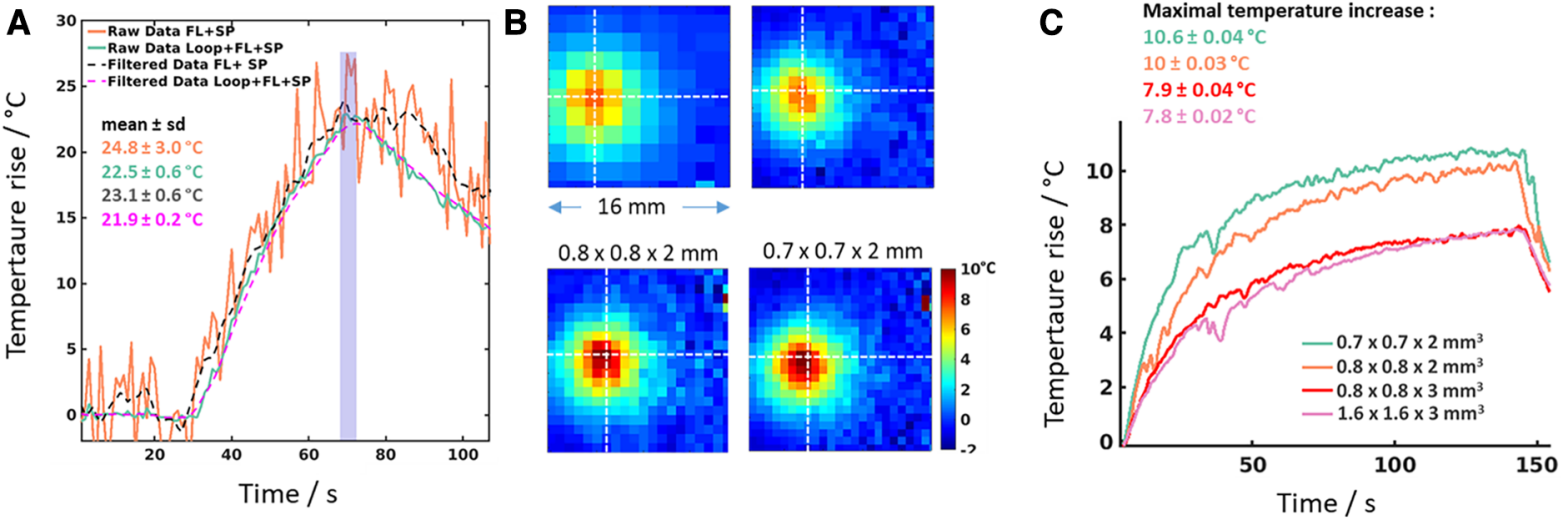
MR-thermometry data on gel phantoms. (**A**) Comparative thermometry during heating during radiofrequency delivery on a gel. The orange curve corresponds to the temperature data obtained when only conventional coils (FL + SP) are used as receivers and without filtering the data (raw data). The black dotted curve represents the same data after low-pass filtering. The green curve corresponds to the raw temperature data obtained when combining the local loop coil with conventional coils (Loop + FL + SP). The pink dotted curve corresponds to the low-pass filtered temperature data acquired with Loop + FL + SP coils. Mean maximal temperature values for each curve were computed over the temporal window displayed in the purple window indicated on the graph. (**B**) Zoomed view (16 × 16 mm FOV) of temperature images acquired with the 2 cm Loop coil at 4 different spatial resolutions (indicated on top of each image in mm) during Laser (975 nm, LuOcean Mini 4, Lumics, Berlin, Germany) heating with identical parameters. The slice was positioned perpendicular to the axis of the optic fiber inserted into the gel. The white dashed cross indicates the pixel with the maximal temperature increase over time. (**C**) Plots of temperature evolution at the hottest pixel for the four conditions are displayed in (**B**) a temporal window of 5 successive acquisitions was used to compute the mean temperature and the temporal standard deviation at the end of the heating experiment (at *t* = 144 s). The resulting maximal temperature (mean ± std) is indicated in the top left corner of the graph for each image resolution.

Figure 3 illustrates the potential of such a coil for MRI thermometry. The graph in Figure 3A compares temperature curves in the same voxel during radiofrequency energy deposition, using conventional receiver coils (original curve in orange and low-pass filtered curve in black dashed line) and conventional coil plus the loop coil displayed in Figure 1A (original curve in green and low-pass filtered curve in purple dashed line). Similar maximal temperatures are measured (ranging from 21.9-24.8°C) although temperature standard deviation values decreased from 3°C (conventional coils) to 0.2°C when the loop coil was activated. Figures 3B,C show temperature data acquired at different spatial resolutions during laser energy deposition in a gel, using only the loop coil for image acquisition. The gain in SNR is exploited to reduce the voxel dimension from 7.68 mm^3^ (1.6 × 1.6 × 3 mm^3^) down to 0.98 mm^3^ (0.7 × 0.7 × 2 mm^3^). In addition to obviously better visualization of the spatial distribution of temperature (Figure 3B), the maximal temperature increase was found to be higher (around 10°C vs. 7.9°C, see Figure 3C) for smaller voxel size, due to reduced partial volume effect. This is a crucial point for the precise characterization of thermal therapy from temperature imaging since the computation of the accumulated thermal dose CEM43 (27) is highly sensitive to small errors in temperature estimates.

Table 1 represents the *Q* factor, detuning efficiency, resonance frequencies *f_+_*, *f_−_*, and the Detuning Offset (DO) obtained in our simulations and VNA experiments. Overall accordance between the DE and resonance frequencies obtained in our simulations and our experiments validates our simulations for more general cases. On-site detuning considered the reference technique, brings an experimental detuning level of 47 dB and a *Q* factor of 49. Although the one-cable remote detuning coil provides a *Q* factor of 55, higher than the 30 of the two-cable remote detuning, its detuning efficiency remains lower (19 compared to 35 dB). This poor detuning efficiency of the single-cable approach is explained by a small detuning offset of 2.3 MHz compared to one of 22.1 MHz obtained with the conventional approach or 9.1 MHz with the double-cable method. As a result, the impedance of such a blocking circuit as well as its detuning efficiency is lower than on-site detuning. Double-cable remote detuning provides a detuning offset of 9.1 MHz, and a detuning efficiency of 35 dB, higher than one-cable remote detuning. However, the second cable and its associated losses explain the lower *Q* value of 30 measured.

**Table 1:** Comparison between experimental and simulated coil characteristics for on-site and remote detuning techniques at *f_0_ *= 64 MHz.

		Experiment			Simulation		
Property	Unit	On-site detuning Figure 2(a)	Single-cable remote detuning Figure 2(b)	Double-cable remote detuning Figure 2(c)	On-site detuning Figure 2(a)	Single-cable remote detuning Figure 2(b)	Double-cable remote detuning Figure 2(c)
Loaded quality factor (*Q*)	—	49	55	30	49	48	26
Detuning efficiency (*DE*)	dB	47	19	35	50	18	34
*f_−_*	MHz	28.9	61.4	52.8	29.0	61.1	53.2
*f_+_*	MHz	86.0	66.2	73.0	85.3	66.3	73.6
DO min(|*f_+/−_*- *f_0_* |)	MHz	22	2.2	9	21.3	2.3	9.6

**Figure 4:**
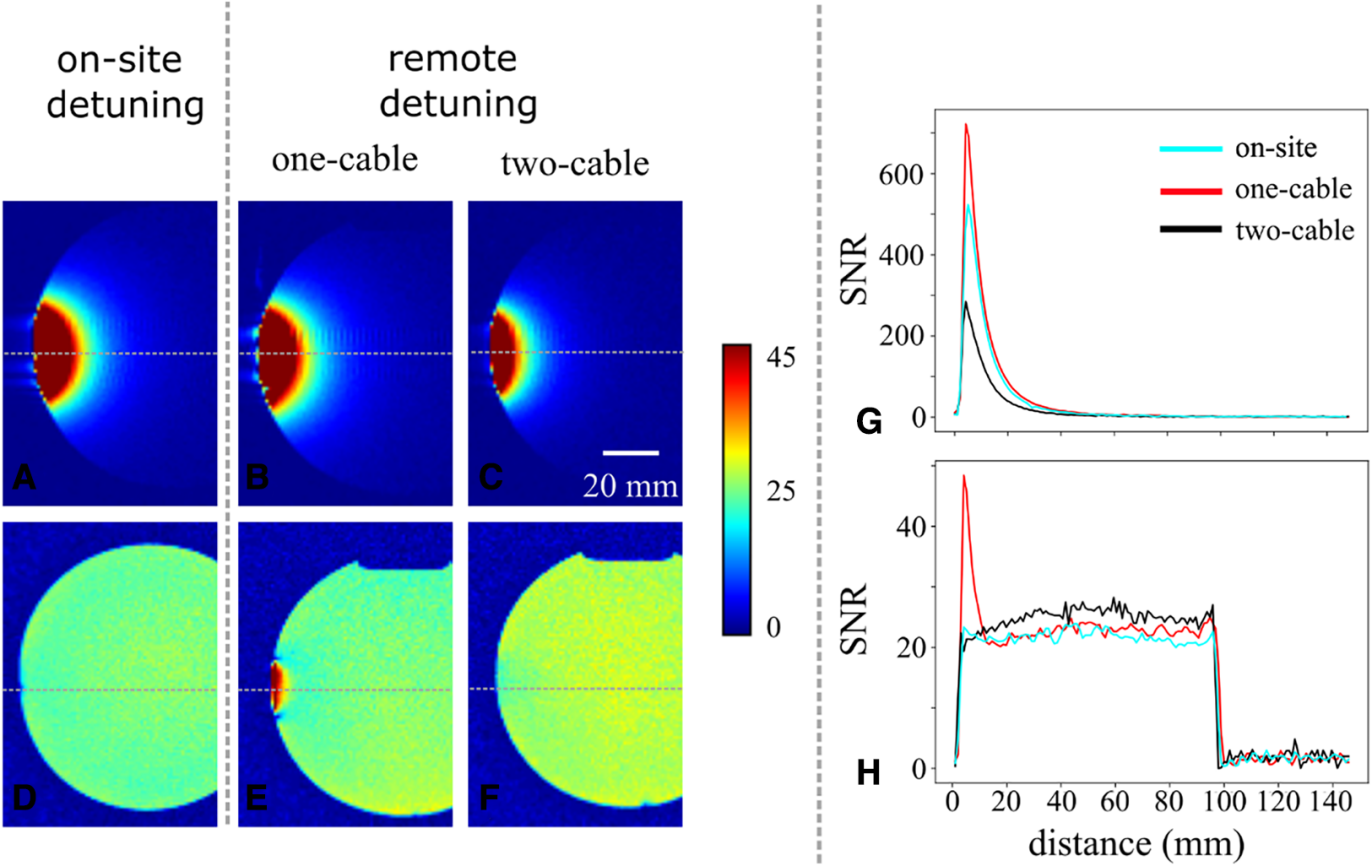
Comparison of sensitivity and detuning efficiency using MRI. At the top row, the SNR maps of three coils detuned with different detuning techniques are compared. (**A**) The classic on-site detuning, (**B**) the single-cable remote detuning, and (**C**) the two-cable remote detuning. For the top row, the integrated body coil was used as the transmit coil, and our fabricated coil was used as the receive coil. At the bottom row, the SNR maps of three coils detuned with different detuning techniques are compared. (**D**) The classic on-site detuning, (**E**) the single-cable remote detuning, and (**F**) the two-cable remote detuning. For the bottom row, the integrated body coil was used as the transmit coil, and a 20-element array head/neck coil was used as the receive coil. During the experiments, our fabricated coils were constantly kept detuned. All maps (top and bottom rows) were obtained by dividing the signal of each pixel by the standard deviation of the noise (rms noise) in each image and are scaled identically (see color bar on the right). Note that for better comparison, only the areas near the coils are shown in the images. (**G**) Intensity profiles on images (**A–C**) at the location indicated by the horizontal gray dotted line. (**H**) Horizontal intensity profiles on images (**D–F**) at the same location.

The sensitivity and detuning efficiency of on-site conventional detuning, and one- and two-cable remote detuning were also assessed by MRI (Figure 4). The SNR maps are shown in (Figures 4A–C). As expected, the sensitivity of the conventional on-site detuning and one-cable remote detuning is almost identical. The sensitivity of these two approaches is superior to the sensitivity of double-cable remote detuning, which allows a penetration depth of about 20 mm compared to a penetration depth around 15 mm for double-cable. Note that a penetration depth of 15 mm is sufficient for intracardiac imaging. The plot in Figure 4G shows the SNR profiles orthogonal to each surface coil in images A, B, and C. All three configurations achieve an SNR greater than 150, with the highest value observed for remote detuning with a single cable. At a distance greater than 10 mm, the intensity profile of this configuration is similar to that of on-site detuning. The SNR profile of dual-cable detuning is approximately 40% lower than that of on-site detuning.

In Figures 4D–F, the detuning efficiency of all three techniques is compared, taking care to keep coils constantly detuned during the experiment. We observed that the detuning efficiency of both double-cable remote detuning and on-site detuning prevented the *B_1_* artifacts very satisfactorily. On the contrary, residual signal close to the coil is observed using the one-cable remote detuning technique, which is illustrative of low detuning efficiency for this implementation. SNR profiles displayed in Figure 4H confirm that two cable remote detuning performs similarly to on-site detuning, while one-cable remote detuning shows a localized overshoot close to the coil.

## 4. Discussion

In this study, we evaluate the potential of using a 2 cm circular coil to improve the spatial resolution of cardiac MR images. Such a diameter was chosen because it could accommodate the size of a human ventricle or atrial cavity and is comparable in size to catheter-based therapeutic devices used clinically. The gain in SNR of our implemented coils reached 35, which was exploited to obtain anatomical images of a beating pig heart with an in-plane resolution below 200 µm. Such a spatial resolution is currently inaccessible with conventional instrumentation, and spatial resolution of clinical cardiac MRI remains at best superior to 1 mm (using 3D acquisitions), limiting precise characterization of the cardiac substrate (Figure 1C). Taking advantage of the high spatial selectivity of the implemented 2 cm surface coils, spatial resolution similar to that of current clinical cardiac MRI can be achieved with a much smaller number of phase encoding steps, thus compensating the lack of parallel imaging capabilities of the proposed configuration. Furthermore, we show in this study that a planar resolution of 170 µm can be obtained in a few minutes on a beating pig heart at 1.5 T. Such a gain in SNR can also be exploited to improve MR-thermometry precision (see Figure 3A) and/or to improve the spatial resolution of temperature images (Figures 3B,C). In the latter case, we illustrate the importance of reducing the partial volume effect that can alter the estimation of the thermal dose, a reference metric commonly used as a therapeutic endpoint in clinical applications of thermotherapy. In the context of interventional cardiac MRI, improving the spatial resolution is a prerequisite to properly monitor ablation in the myocardium, particularly for the atria whose wall thickness ranges between 2 and 5 mm. Temperature data reported in Figures 3B,C still needs to be optimized regarding spatial resolution, although this was not the main objective of this study. However, considering the temperature standard deviation reported here (0.04°C), further reduction of voxel dimension appears possible while maintaining acceptable precision of temperature estimation (around 1°C), as reported previously during MRI-guided catheter ablation in the ventricle using conventional receiver coils (16, 28, 29). In the perspective of designing an MR-compatible catheter equipped with a receiver coil, we strongly focus on active detuning techniques in the present study. Conventional on-site detuning provides the best overall detuning and sensitivity performance. However, in exceptional cases where the available space is limited, or exploiting a DC to bias the diode (or powering a miniaturized pre-amplifier) represents a risk for the patient (e.g., in interventional cardiovascular MRI), implementation of on-site detuning could become technically challenging, expensive, and hazardous.

In the primary implantation of remote detuning (one cable), a single coaxial cable carries the signal and detunes the receiver coil. The sensitivity in this approach is similar to classic on-site detuning. However, the detuning efficiency obtained by this method may be insufficient to prevent *B*_1_ artifacts, as illustrated in Figure 4E. Implementing the two-cable remote detuning provides higher detuning efficiency than the method proposed by Edelstein (19 vs. 35 dB) and in Figure 4F. The freedom to choose the coupling capacity value explains this improvement in DE since there is no longer the constraint on its value to be matched to the receiving channel.

However, due to the second cable and its associated losses, a lower Q of 30 was measured, slightly decreasing the sensitivity of the two-cable approach (penetration depth decreases by 25% and SNR by 40%). This reduction in sensitivity strongly depends on the dominant origin of loss in the system. For circular loops, the question of the dominant source of loss as a function of the coil radius has been addressed by Kumar et al. (30). When tissue noises are the dominant noise, the sensitivity of two-cable remote detuning could be the same as conventional detuning or one-cable remote detuning. This configuration will no longer be the case if the coil noise becomes dominant, as in the phantom experiments presented in this paper, since our coils are small enough, and the losses in the coil components are significant.

Simulations and experiments showed excellent agreement with a deviation of around 10%, allowing coil designers to optimize coil components by simulation before implementation. We show that effective remote detuning can be achieved without injecting DC into the patient and by positioning the required components (the PIN diode, the decoupling inductor, and the RF choke) outside the body. Adding a second cable for detuning increases the device's size by less than a millimeter using available cables. For single-use devices, remote detuning also allows a significant reduction in construction costs.

### 4.1. Study limitations

Only rigid circular coils of fixed dimensions (2 cm diameter) were implemented in this study. In the context of interventional MRI, a deployable MR coil should be embedded in a catheter or guided to the desired heart cavity using a sheath. Several technical solutions were reported in the literature (10, 11, 12) but with limited experimental validation. Such technologies could not be implemented in the context of this study, which was centered on (1) evaluating the potential of a local coil for high-resolution anatomical cardiac MRI and MRI thermometry, and (2) providing technical solutions for remote active detuning. Once implemented, the effective gain in SNR should be quantified since noise figures and associated losses may vary when the coil is inside a cardiac cavity, using a similar experimental setup of ex vivo beating swine hearts, before envisioning *in vivo* evaluation.

Remote active detuning was achieved using either one or two cables. The presence of long cables inside an MRI may result in hot spot generation at the cable's end, particularly due to common mode currents circulating in the external conductor of coaxial cables. Such a risk was not analyzed here and requires further investigation when a final prototype of a deployable MR receiver embedded in a catheter will be implemented. For this purpose, local temperature probes can be inserted to monitor potential heating at expected hotspot locations. Alternatively, MR thermometry can be used to map temperature changes around the cables, as recently proposed (31). However, the risk of creating hotspots around the cables can be attenuated by incorporating the cable(s) in a catheter/sheath equipped with an external metallic mesh that acts as a Faraday cage. A recent publication proposed inserting balun traps at regular distances on such a sheath to alleviate the risks of cable heating during MRI acquisition (10).

## 5. Conclusion

In this study, we report high-resolution anatomical images of a beating heart and show interest in small-dimension detectors. Remote detuning techniques of a receive-only MRI coil have been investigated. The principles of single cable and two cables remote detuning were presented. In addition, the sensitivity and detuning performance of these methods in RF bench and MRI experiments were compared with classic on-site detuning. We also validated simulation by RF bench measurements to help coil designers to predict coil performances before their effective implementation. From an interventional cardiac MRI perspective, the two-lead remote detuning configuration reduces the number of electronic components located on the coil while avoiding carrying DC current inside the body. This study demonstrates that high-quality images can be obtained on clinical MRI to improve the diagnosis of heart disease and better guide interventions.

## Data Availability

The raw data supporting the conclusions of this article will be made available by the authors, without undue reservation.
