# Supplementary material for: Remotely detuned receiver coil for high-resolution interventional cardiac magnetic resonance imaging
Source: Front Cardiovasc Med. 2023 Oct 30;10:1249572. doi: 10.3389/fcvm.2023.1249572 (PMC10643167; doi:10.3389/fcvm.2023.1249572)
Supplement: Supplementary file 1 [file Datasheet1.pdf]

Supplementary Material

Remotely detuned receiver coil for high-resolution interventional cardiac Magnetic Resonance Imaging

Sina Marhabaie, Marylène Delcey, Dounia El Hamrani, Fanny Vaillant, Jean-Christophe Ginefri, Valéry Ozenne, Emma Abell, Marie Poirier-Quinot, Bruno Quesson*.

*** Correspondence:** Bruno Quesson: bruno.quesson@u-bordeaux.fr

1 Supplementary Figures

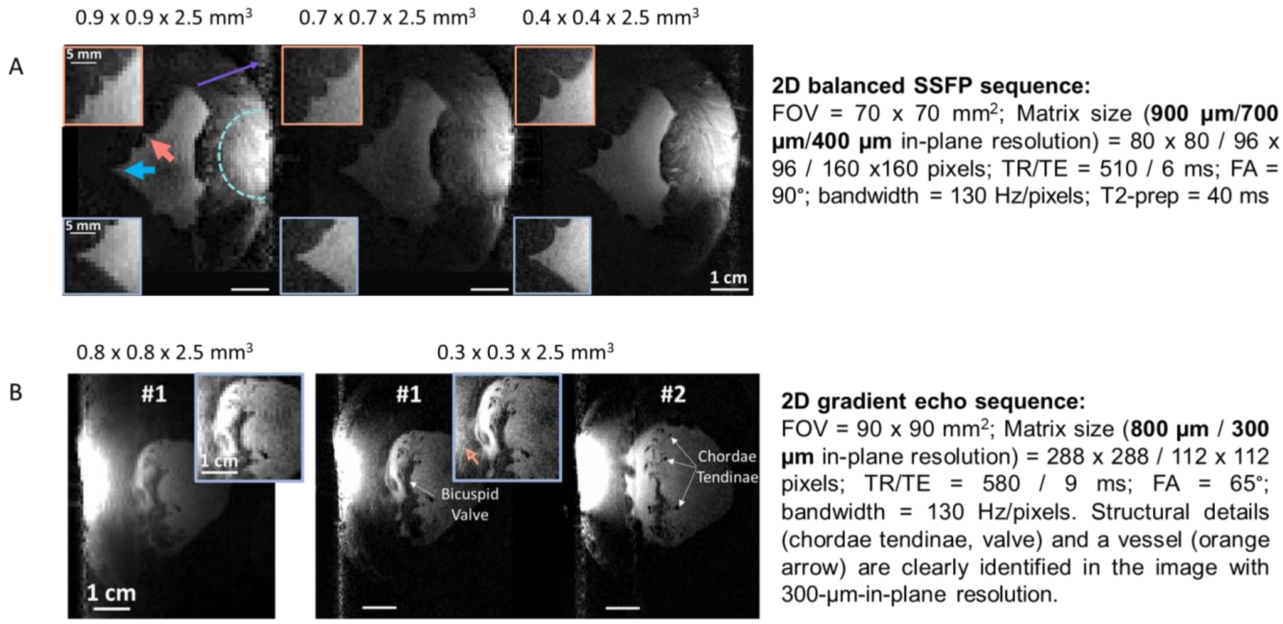

Figure S1. Images obtained at different spatial resolutions on two different ex vivo beating hearts from swine.

Acquisition sequences were triggered on the heartbeat (1 k-space segment acquired per cardiac contraction) and imaging parameters are indicated on the right. (A) The total acquisition time was 2 min, 2 min 27 s, and 4 min for the in-plane resolutions of 900 µm, 700 µm, and 400 µm, respectively. Orange and blue arrows indicate the position of the zoomed inserts in each image, illustrating the increased definition of the endocardial wall structure at 400 µm in-plane spatial resolution. The purple arrow indicates a residual motion artifact due to a small blood leak near a sutured pulmonary vein. The green dashed ellipse highlights the receiver coil sensitivity. In the inserts, contrast windowing has been adjusted for better visualization. (B) The total acquisition time was 2 min 10 s (800 µm in-plane

resolution) and 5 min 20 s (300 µm in-plane resolution), respectively. Images were acquired at the same location at two different in-plane resolutions. Numbers on the images indicate the slice number of the acquisition sequence. Inserts show a zoomed view with a small vessel visible at 300 µm in-plane resolution (orange arrow).

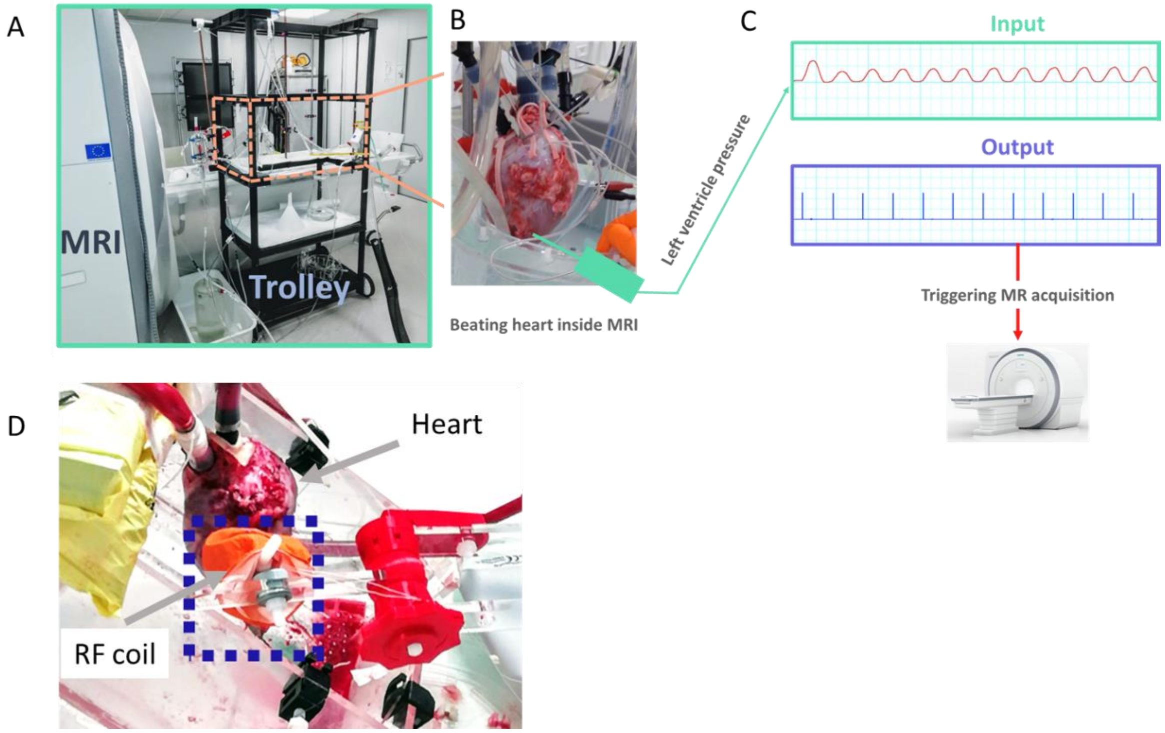

Figure S2. Description of the experimental setup for MR-imaging of a beating heart from pig.

(A) Photograph of the MR-compatible beating heart setup.
(B) Photograph of a beating pig heart connected to the perfusion setup
(C) Continuous intraventricular pressure sensor recordings allowed generating a TTL signal to trigger MRI acquisitions.
(D) Photograph of the holder to maintain the 2 cm coil (protected by an orange glove) in contact with the beating heart.

2 APPENDIX

2.1 Classic Detuning and Coupled Resonators

The blocking circuit is usually considered a parallel resonant circuit with a large impedance at its resonance frequency, efficiently impeding the circulation of current in the receive coil. One may understand detuning more accurately by resorting to the general properties of two coupled resonant circuits. A typical implementation of classic detuning is presented in Figure S3 (A). The system consists of two coupled sub-circuits (the receive coil and the blocking circuit), that resonate at the same frequency, which $f_0 = \omega_0/2\pi$ is the Larmor frequency.

$$L_R\ \omega_0 - \frac{1}{\omega_0}\left(\frac{1}{C_T} + \frac{1}{C_C}\right) = L_D\ \omega_0 - \frac{1}{\omega_0\ C_C} = 0$$

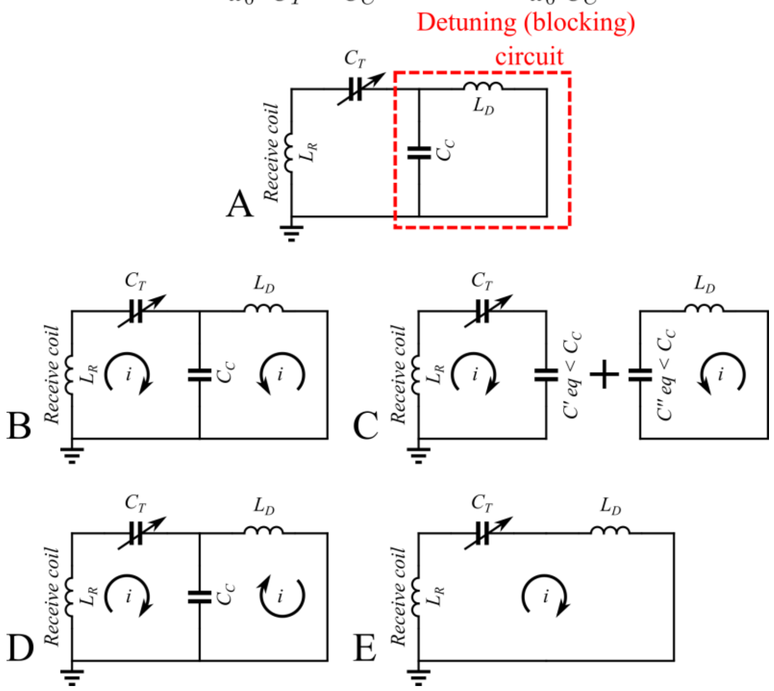

Figure S3: Circuit analysis of coupled resonators

The circuit is composed of L_R the inductance of the receive coil, L_D the inductance of the detuning coil, C_T the tune capacitance, and C_C the coupling capacitance used to couple the two circuits. The current circulation in the high-frequency mode (f_+) and its equivalent circuit are depicted in (B) and (C), respectively. Also, (D) and (E) represent the current circulation in the low-frequency mode (f_-), and its equivalent circuit, respectively.

It turns out that for the system shown in Figure S3A, there are two resonance modes with two different frequencies given by:

$$f_{\pm}^2 = \omega_{\pm}^2/4\pi^2 \;\; = \omega_0^2/4\pi^2 \left[1 \pm \sqrt{\frac{L_D}{L_R}}\right] = \omega_0^2/4\pi^2 \left[1 \pm \frac{1}{\sqrt{1+\frac{C_C}{C_T}}}\right] \quad (1)$$

where f_+ and f_- are the two new resonance frequencies, respectively high and low, of the detuned receive coil, L_R is the inductance of the receive coil, L_D is the inductance of the detuning coil in the blocking circuit, C_T is the tune capacitance, and C_C is the coupling capacitance used to couple the receive coil to the blocking circuit. In Figure S4, f_+ and f_- of a typical coil in the detuned state (orange), the single resonance frequency, f_0, of the same coil in the non-detuned state (green), as well as the associated detuning efficiency (*DE*, red) are depicted. This figure has been obtained using a standard double-loop probe S_{12} measurement [4], [17]. In this article, the offset of the new frequencies from the Larmor frequency is referred to as the detuning offset, defined as $DO = min(|f_{+/-} - f_0\,|)$.

DE is related to the detuning offset value. A small *DO* generally leads to a low *DE* (inefficient detuning). According to Eq.(1), the smaller the ratio C_C/C_T, the larger the detuning offset. Provided that the *DO* is sufficient (f_- and f_+, both are sufficiently far from f_0), the system represents a high impedance at f_0 (like any other resonant system far from its resonance frequency). The current that circulates in the coil during the transmission phase will be considerably reduced. In ordinary cases, by choosing the correct values for L_R, L_D, C_T, and C_C, one can obtain a sufficient detuning offset and hence an efficient detuning.

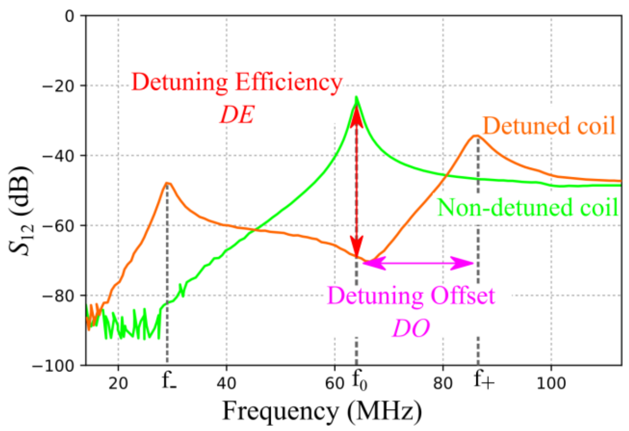

Figure S4: S12 curves in tuned/detuned conditions and measurement of the Detuning Efficiency (*DE*) and Detuning Offset (*DO*)

Typical S_{12} curve obtained by a double-loop probe when the reference coil used in this study is not detuned (green curve) and or detuned (orange curve). The single resonance (f_0 =64 MHz) observed when the coil is tuned splits into 2 resonances (29 and 86 MHz) when detuning is activated. The Detuning Efficiency (depicted by a red double-headed arrow) is the difference between the orange and the green curves at the Larmor frequency (here 45 dB). *DO* (depicted by pink double-headed arrows) is defined as the $min(|f_{+/-} - f_0|)$.

2.2 Calculation of resonance frequency of a remotely detuned coil

To calculate the two resonance frequencies of a remotely detuned receive coil consider the circuit shown in Figure S5. There are two modes shown by green and brown arrows where the current of the left sub-loop, i_L, and the current of the right sub-loop, i_R, circulate in opposite directions.

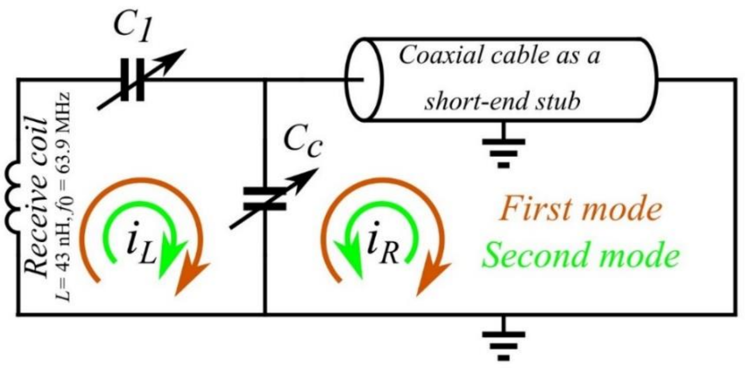

Figure S5: Schematic diagram to illustrate the calculations of the two resonance frequencies of a remotely detuned receive coil. Two modes correspond to different directions of current circulation. Each mode corresponds to a different resonance frequency.

One may write the voltage equation for this circuit that leads to two coupled differential equations and solve the equations to find the two resonance frequencies. A more elegant approach is to assume an alternating voltage and consider the complex impedance of each element. In this way, instead of a differential equation, we obtain an algebraic equation for each mode, the root of which is the resonance frequency of that mode. This approach has been used to obtain the resonance frequencies of two coupled LC circuits (N. H. Fletcher and T. D. Rossing, 'Coupled Vibrating Systems', in The Physics of Musical Instruments, New York, NY: Springer New York, 1998, pp. 102–132; Eq. 1 was also derived using this approach), and we exploit it here to get the resonance frequencies of the LC circuit connected to a piece of coaxial cable, Figure S5. The complex impedance of an inductor is $Z_L = j\, L\, \omega$, that of a capacitor is $Z_C = \frac{1}{j\, C\, \omega}$, that of a short-end stub is $Z_{short} = j\, Z_0\, tan\left(\frac{\omega}{v}\, l\right)$, where $j^2 = -1$, and Z_0 is the characteristic impedance of the stub (50 Ohms here), l its length, and v the propagation velocity of the stub. Considering these complex impedances, we write the voltage equation in the left sub-loop for the first mode, in which the i_L and i_R have opposite phases.

$$[\omega_0^2 - \omega^2]\, i_L - \frac{1}{L\, C_C} i_R = 0 \tag{2}$$

with $\omega_0^2 = \frac{1}{L}\left[\frac{1}{C_1} + \frac{1}{C_C}\right]$.

The second equation is obtained by writing the voltage equation in the right sub-loop for the first mode:

$$\left[\frac{1}{\omega\, C_C} - Z_0\, tan\left(\frac{\omega}{v}\, l\right)\right] i_R - \frac{1}{\omega\, C_C} i_L = 0 \qquad (3)$$

considering Eqs. (2) and (3), we can write:

$$1 - \omega\, C_C\, Z_0\, tan\left(\frac{\omega}{v} l\right) = \frac{1}{[\omega_0^2 - \omega^2]\, L\, C_C} \qquad (4)$$

The two resonance frequencies of our circuit are the roots of Eq. (4). This equation can be solved either numerically, or by graphical methods. For pedagogical reasons, here, we choose the visual approach. Either parts of Eq. (4) are plotted in Figure S6. Figures S6A and S6B, correspond to the circuits presented in Figures 2B and 2C, with C_C values of 833 pF and 133 pF, respectively. Around ω_0, $\frac{1}{[\omega_0^2-\omega^2]\, L\, C_C}$ varies sharply (blue curve in Figure S6) and far away from ω_0, $1 - \omega\, C_C\, Z_0\, tan\left(\frac{\omega}{v} l\right)$, behaves like a line, the slope of which varies with C_C (orange curve in Figure S6). The roots of Eq (4), the cross points on the blue and the orange plot, are mainly determined by the behavior of $1 - \omega\, C_C\, Z_0\, tan\left(\frac{\omega}{v} l\right)$, which means the smaller the C_C is, the larger the detuning offset *DO,* and the more efficient detuning (the larger *DE*)

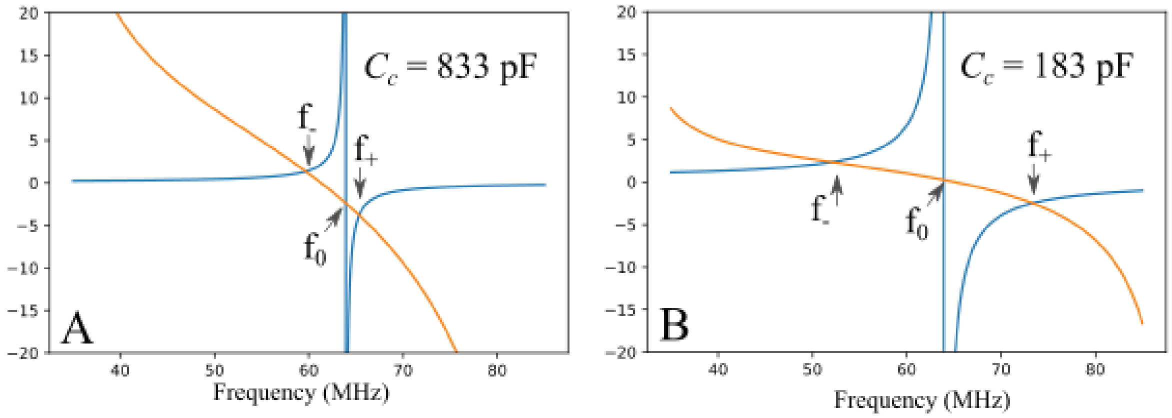

Figure S6: Graphical solution of Eq (4)

Both parts of Eq. (4) are plotted as a function of frequency, $\frac{1}{[\omega_0^2-\omega^2]\, L\, C_C}$ in blue and $1 - \omega\, C_C\, Z_0\, tan\left(\frac{\omega}{v} l\right)$ in orange. The roots of the equation, i.e. f_- *and* f_+ the resonance frequencies of the circuit shown in Fig. S4, are the crossing points of the two curves. In (A), a large value for C_C of 833 pF is assumed, which results in a small detuning offset. In (B), a small C_C value of 133 pF is assumed, resulting in a large detuning offset.
